# The South

**Published:** 1892-04

**Authors:** 


THE SOUTH. A journal of Southern and Southwestern Progress. Published monthly by "The South" Publishing Company, No. 22 College Place, New York. Subscription price, $1.50 per year, in advance.

This journal, as its name indicates, gives most interesting and valuable accounts of the industrial development of the South. Many instructive facts are found under the headings of its various departments, such as Southern Industrial Notes, Railroads, Electricity, Construction, and Machinery. An article in the January number, entitled "What Free Trade would do for the South," gives an idea of the political stand taken by this journal.

